# Detecting the Six Polytypes of Five‐Layer Graphite

**DOI:** 10.1002/adma.202509947

**Published:** 2025-09-02

**Authors:** Nirmal Roy, Shaked Amitay, Simon Salleh Atri, Harel Kabla, Oren Ben Moshe, Moshe Ben Shalom

**Affiliations:** ^1^ School of Physics and Astronomy Tel Aviv University Tel Aviv 6997801 Israel

**Keywords:** electric polarization, graphitic polytypes, Raman spectroscopy, surface potential

## Abstract

Graphene layers can assemble in two shifted metastable positions per interface, leading to eight possible structural arrangements in five‐layer graphene, six of which correspond to distinct periodic crystals. These polytypes exhibit diverse symmetries, interlayer electronic hybridization, van der Waals adhesion, and optical responses. Arrangements lacking inversion [I] and mirror [M_z_] symmetries host intrinsic polarizations, while those with sufficiently flat electronic bands display orbital magnetization, unconventional superconductivity, and anomalous fractional quantum Hall states. Here, a detailed comparative study of these polytypes is presented, examining their Raman scattering spectra, second‐harmonic optical emission microscopy, and surface electric potential maps. Each configuration is deterministically identified, its natural abundance is quantified, and its relative stability is assessed. Understanding these stacking‐dependent properties is a critical step toward the development of multiferroic devices that leverage sliding transitions between structural configurations.

## Graphical abstract

The six possible periodic crystals—so‐called polytypes of Penta layer graphene are identified using Raman, second harmonic, and electric surface potential measurements. The measurements reveal the polytypes’ abundance, relative stability, and annihilation dynamics, crucial for future multiferroic SlideTronic applications.

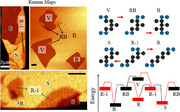

## 1 Introduction

**Figure 1:**
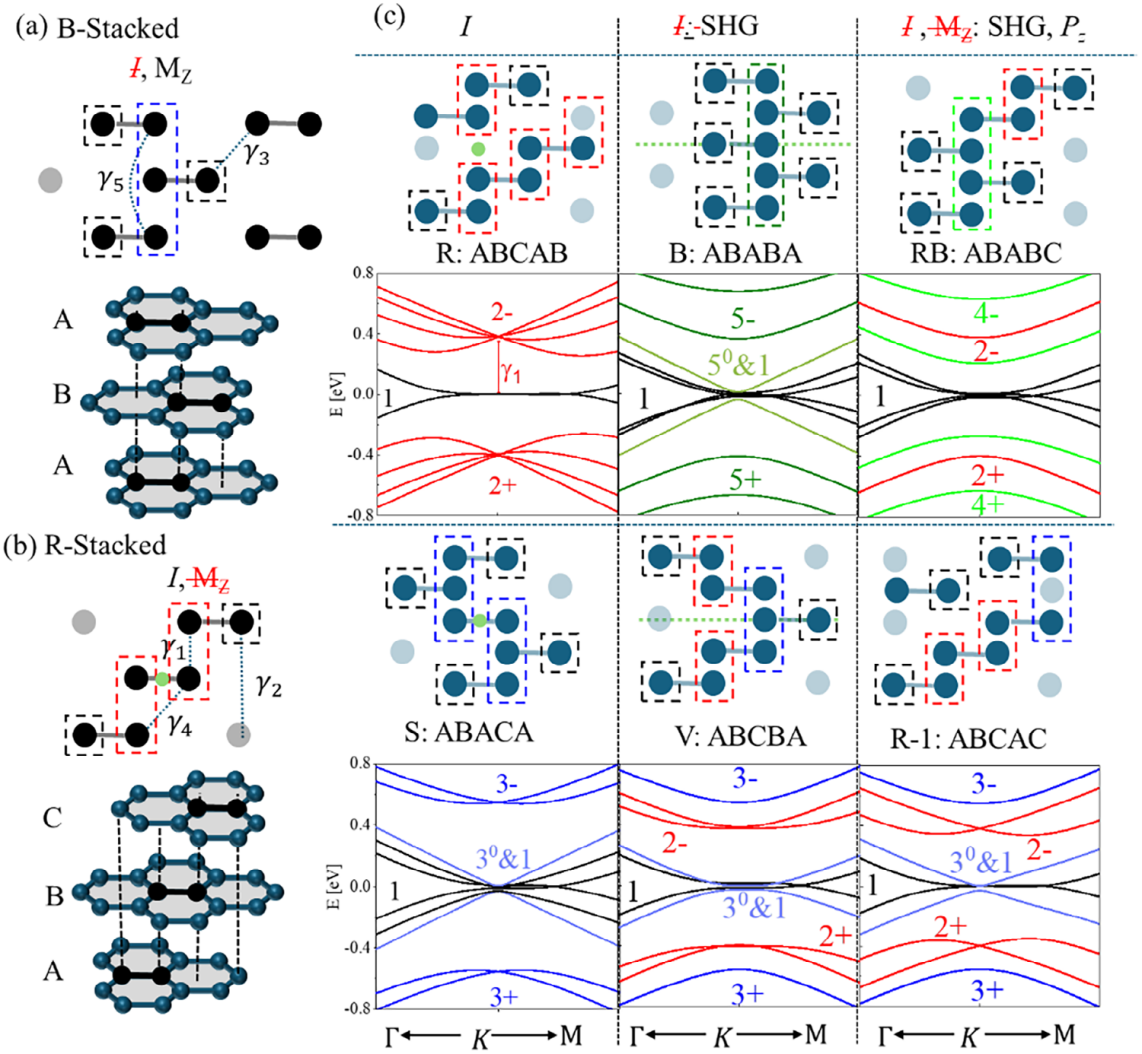
Illustration of Penta‐layer polytypes a,b) stacking configuration, illustration of tri‐layer Bernal (B) and Rhombohedral (R) graphene. Unit cell atoms are colored in black. Dashed lines mark the leading hopping parameters. c) Unit cell and electronic band structure of all Penta‐layer polytypes. The corresponding inversion [I] and mirror‐plane [M] symmetries are shown by a green point/dashed line. The red/blue/green/dark green electronic band corresponds to di/tri/tetra/pentamers states, as marked in the unit cell by a dashed frame. The K space range is Γ→Κ/10 (Κ→M/5).

A pair of graphene layers can stack in two high‐symmetry arrangements, forming metastable periodic crystals. In a stack of N layers (with N‐1 interfaces), this results in a total of 2^N‐1^ possible configurations. Of these, 2^N‐3^ + 2^(N‐4)/2^ and 2^(N‐3)^ + 2^(N‐3)/2^ represent inequivalent crystal structures for even and odd, respectively.^[^ ^1^ ^]^ For instance, tri‐layer graphene (N = 3) has two metastable polytypes (**Figure** **1**a,b), while tetralayer graphene (N = 4) has three. Constructed entirely of carbon atoms, each with a single available electron, these elemental polytypes provide a unique platform for studying the structural influence on material properties. Additionally, they enable intuitive and relatively accurate tight‐binding models for electronic band structures, which govern many physical characteristics.^[^ ^2^, ^3^ ^]^

To characterize each polytype, a periodic unit cell is identified by selecting two atoms per layer. In trilayers, the unit cell consists of six orbitals, which relax into either a Bernal (B) or Rhombohedral (R) structure (Figure 1a,b). Notably, perfectly aligned graphene layers are energetically unfavorable compared to shifted configurations. Consequently, all metastable graphitic interfaces exhibit eclipsed stacking in which half the atoms align while the other half are positioned over an empty hexagon center.^[^ ^4^, ^5^ ^]^

Next, interlayer electronic interactions must be considered (see dotted yellow lines in Figure 1a,b). The in‐plane hopping energy γ_0_ ≈ 3.16 *eV* determines the monolayer bandwidth, while the stacking‐dependent vertical overlap γ_1_ ≈ 0.39 *eV* governs energy shifts between eclipsed atoms (see Figure S9, Supporting Information). Hopping within these eclipsed pairs (dashed red frame in Figure 1b,c) creates bonding and antibonding dimer states, shifting the corresponding energy levels by ± γ_1_ away from the Fermi energy (see red bands in Figure 1c, corresponding to states localized on the three dimers of this polytype). More generally, at the K, K’ corners of the Brillion zone, these energy shifts follow the relation

(1) $$E_{N,n}=2\gamma_{1}\cos\left(\frac{n\pi }{N+1}\right)$$

where N is the number of eclipsed orbitals and $n\;\varepsilon [1,{\left(\frac{N}{2}\right)}_{int}]$.^[^ ^6^, ^7^ ^]^ For example, trimer states (dashed blue frame) are shifted by ≈0.55 eV, (blue bands of the polytypes in the second row), while tetramer states (light green frame) are shifted by ± 0.24, ± 0.63 eV, and the pentamers bands (dark green) shift by ± 0.39, ± 0.68 eV. Conversely, atoms in hollow sites (dashed black) contribute additional bands at the Fermi energy, which disperse as ≈  *q* ^*M* + 1^, where *q* is the crystal momentum measured from K, K’, and M represents the number of γ_1_‐mediated hops to the nearest monomer. Furthermore, hybridization between monomers and odd trimers (3^0^ and 1) or pentamers (5^0^ and 1) gives rise to dispersive bands crossing the Fermi energy (light blue and light green bands). This straightforward method of counting eclipsed clusters provides an intuitive understanding of stacking‐dependent electronic dispersion and material properties.

Energy shifts on the scale of γ_1_ modify the infrared (IR)^[^ ^7^, ^8^ ^]^ and Raman response,^[^ ^9^, ^10^, ^11^, ^12^, ^13^ ^]^ while the flat dispersions with diverging density of states result in correlated electronic phases such as superconductivity,^[^ ^14^ ^]^ orbital magnetism,^[^ ^15^, ^16^ ^]^ and anomalous quantum Hall states.^[^ ^17^, ^18^ ^]^ Beyond energy shifts, the interlayer hybridizations modify the [I] (r→‐r) and [M_z_] (z →‐z) symmetries, thereby redistributing charge across orbitals. Polytypes that break [I] exhibit second harmonic generation (SHG), whereas those that break both [I] and [*M_z_*] develop intrinsic out‐of‐plane electric polarizations. These symmetry‐dependent properties have been leveraged to distinguish polytypes and their inequivalent configurations. For example, B and R trilayers exhibit distinct SHG signals,^[^ ^19^ ^]^ while the two configurations of the polar polytype in tetralayers that break [I] and [*M_z_*] were detected using electric surface potential measurements.^[^ ^1^ ^]^

This work investigates penta‐layer polytypes, categorized by their symmetry properties: two preserve [I] symmetry, two break [I], and two break both [I] and [M_z_] symmetries (Figure 1c). Each polytype is labeled in Figure 1c according to its unit cell arrangement. In R polytypes, also known as ABCAB stacking, successive interlayer shifts occur in the same direction, while B polytypes (ABABA) exhibit alternating shifts (Figure 1a,b). We refer to the intermediate polytypes as RB (ABABC) and R‐1 (ABCAC), while the remaining ABACA and ABCBA configurations are denoted as S and V, respectively—names reflecting their unit cell shapes and symmetry features. With growing interest in penta‐layer graphene, reports have emerged of fractional quantum anomalous Hall state,^[^ ^20^, ^21^ ^]^ multiferroic orbital response,^[^ ^22^ ^]^ layer‐polarized ferromagnetism,^[^ ^23^ ^]^ and the tunable switch between these polytypes in superlubricant arrays.^[^ ^24^, ^25^ ^]^ These findings highlight five‐layer graphene as a promising system for tailoring material properties, with broad potential for future technological applications.

## 2 Device Preparation

Our Penta‐layer graphene samples were exfoliated from natural graphite crystals onto 90 nm‐thick Si/SiO_2_ wafers. We confirmed the number of layers by optical contrast, Raman intensity ratio, and Atomic Force Microscopy (AFM) measurement.^[^ ^26^ ^]^ To ensure repeatability, we maintained pristine surfaces during the measurement and an external doping level below 5 × 10^12^ cm^−2^ by cleaning the surfaces with an AFM tip and maintaining an inert environment during the measurements. In some cases, these required repeated cleaning.

## 3 Raman Spectroscopy

**Figure 2:**
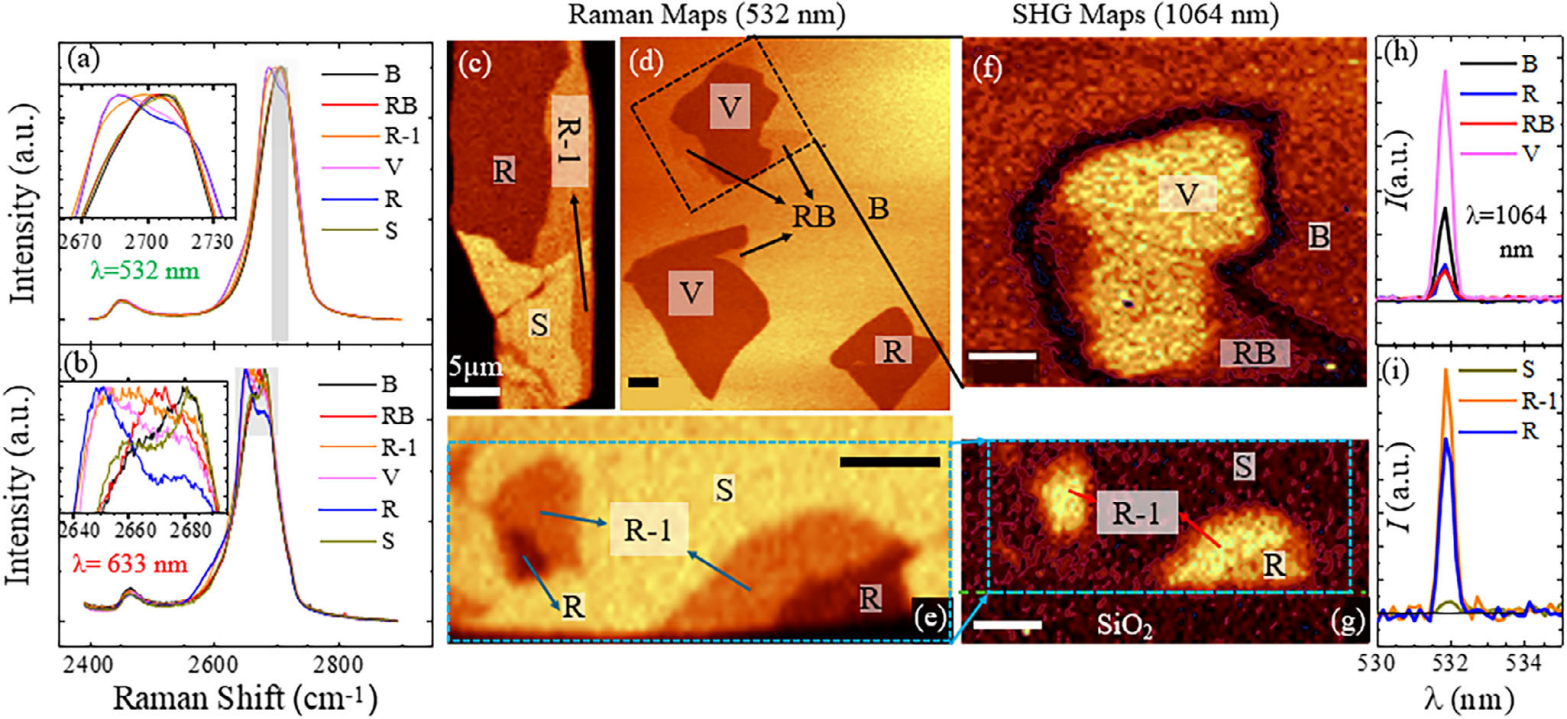
Raman and SHG map a,b) 2D Raman spectra of all six polytypes obtained with green (532 nm) and red (633 nm) lasers. Inset highlights the distinct features of each polytype. c–e) Integrated intensity map of 2D peaks (in the range of 2690–2720 cm^−1^) using 532 nm laser, revealing polytype distribution across three graphene flakes. f,g) SHG intensity map of selected regions. h,i) Average SHG intensity of polytype regions.

Raman spectroscopy of more than 100 penta‐layer flakes was captured and analyzed using a WITEC alpha300 Apyron confocal microscope under green (532 nm) and red (633 nm) laser excitations. At each pixel, we collect a full scattering spectrum (**Figure** **2**a) using green laser illumination and plot a color map representing the number of photons collected in a specific energy range. Figure 2c–e shows filtered maps of representative flakes in the range 2690–2720 cm^−1^ (gray shaded filter in Figure 2a). The latter filter creates sufficient contrasts to distinguish three stacking domains in each flake, although the line spectra in Figure 2a seem to overlap in some cases. Conversely, line‐spectra measurements using a red rather than a green laser provide a clear distinction of the six polytypes, as shown in Figure 2b. The red illumination, however, reduces the count rates substantially, which limits practical mapping of the entire sample. These distinct line‐spectra identifications hold in all samples measured, provided the doping level is kept below 5 × 10^12^ cm^−2^ (which requires the removal of any surface absorbents).

## 4 Identifying Polytypes

After identifying six distinct Raman spectra, we turn to match each spectrum to a particular polytype (see a summary of the full procedure, Table S1, Supporting Information). First, we identify the B and R polytypes as the black and blue spectra based on their similarity to the tetra‐layer polytypes and their abundant appearance.^[^ ^9^ ^]^ We find that penta layers B and R polytypes appear in ≈60% of the flakes. The next spectrum to distinguish is the S polytype, which is the only polytype other than R that preserves [I] and hence exhibits a minute SHG response. Figure 2f,g shows SHG intensity maps of two samples taken at a 1064 nm laser illumination. The average SHG intensity of each domain is presented in Figure 2h,i, respectively. The S domain, having no SHG, exhibits the dark yellow curve in the Raman spectra of Figure 2a,b. We note the residual SHG response of the R polytype, which is absent in the S polytype, although both preserve [I]. The difference is attributed to a residual interaction with the substrate that breaks the symmetry and is more pronounced in the R phase. To see why, we point to the real‐space position of the Fermi energy states in these two polytypes shown in Figure 1c. In the R phase, the two flat bands reside on two surface monomers and are susceptible to the nearby substrate, while the S phase divides the Fermi‐energy states between six bands that spread over the five layers. This surface wave function distribution of the R phase makes it more sensitive to contaminants, further inducing SHG responses.^[^ ^27^ ^]^

The three remaining spectra are the pink and orange curves that resemble the blue R spectrum, and a red spectrum that resembles the black B spectrum (see the curves overlap in the range 2640–2690 cm^−1^ at the left part of the filter in Figure 2a,b). We identify the former two as the R‐1 and V polytypes that exhibit similar band structures with three monomers, two dimers, and one trimer (Figure 1c). Conversely, the RB phase with only one dimer shows reduced intensity in the ∼2640 cm^−1^ energy range.

## 5 Stability and Annihilation Dynamics

**Figure 3:**
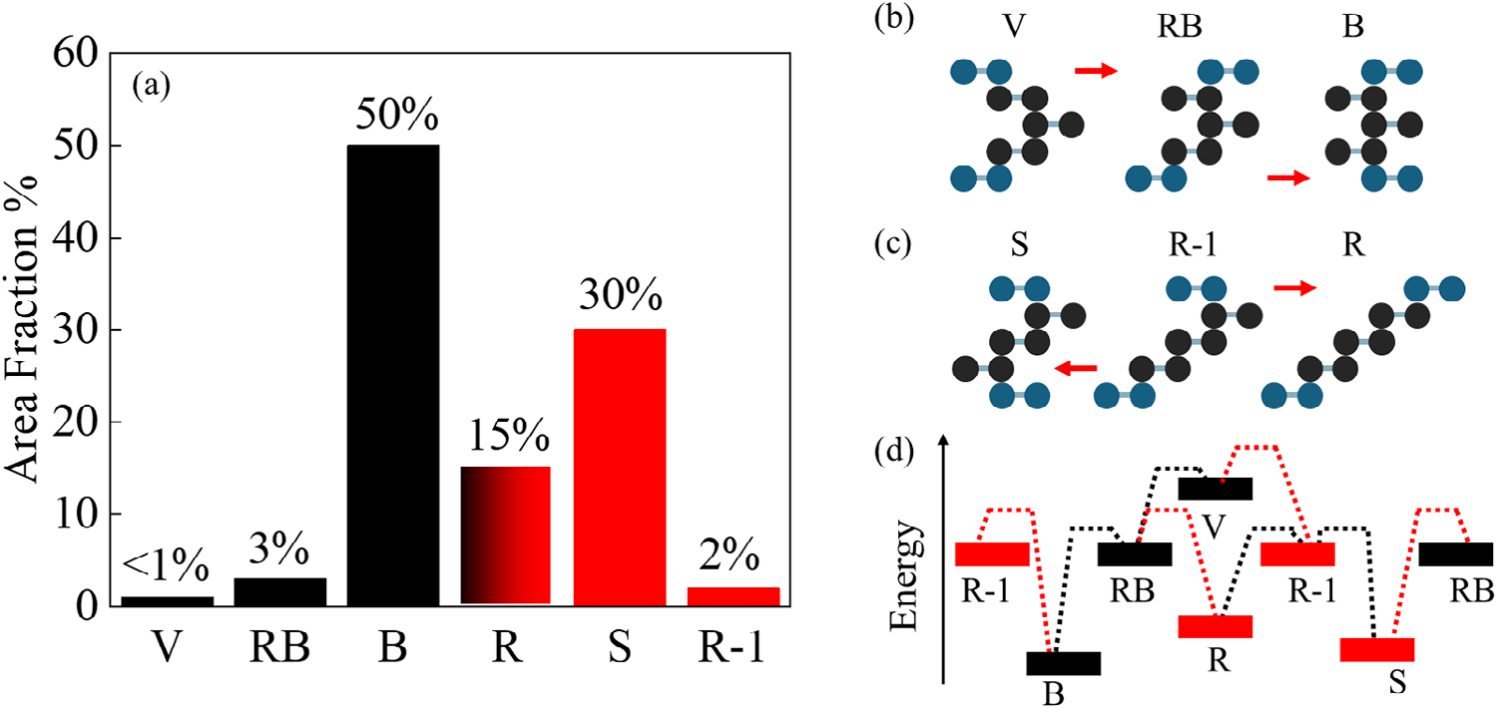
Abundance and annihilation dynamics. a) Histogram of the area fraction observed in exfoliated Penta‐layer graphene. Black and red colors indicate two groups that tend to appear together. b,c) illustrate a single‐step transition in the V ↔ RB ↔ B and the S ↔ R‐1 ↔ R groups, with a shift occurring in only one surface layer. d) A simplistic model of relative stability. Dotted black/red lines mark a transition by shifts within the outer/inner interfaces with a lower/higher energy barrier, respectively.

To further distinguish between R‐1 and V, we turn to their relative stabilities and switching dynamics. The bar histogram in **Figure** **3**a shows the area fraction that we find for each polytype, after analyzing ≈100 flakes, just after exfoliation and Raman mapping. B and S polytypes are most abundant, covering ≈80% of the penta‐layer flakes, while the R phase covers 15% of the area. The V, RB, and R‐1 phases, on the other hand, are relatively rare and tend to annihilate easily into the more stable B, R, and S polytypes over time and during the measurements, as shown in Figure S1 (Supporting Information). Interestingly, V, RB, and B tend to appear together as part of a given flake, or alternatively, the flake contains R‐1, R, and S. Transitions within these two groups (noted by black and red bars in Figure 3a respectively) involve sliding along one of the outer interfaces (top or bottom) without changing the stacking of the three middle layers with two inner interfaces (Figure 3b,c). The rareness of sliding transitions along inner interfaces indicates higher energies of boundary strip dislocations at these interfaces and larger transition barriers. Thus, the overall abundance of a given polytype depends on its relative adhesion potential and on the intermediate transition barrier to the other more stable phases.^[^ ^28^ ^]^

To describe the annihilation dynamics observed, we propose a switching diagram between the phases in Figure 3d. At the center of the diagram, we place the RB and R‐1 phases that break M_z_ since each shift along any of their interfaces leads to another polytype. The other B, R, S, and V phases, on the other hand, are mirror symmetric and switch into two phases only by an inner or an outer shift. Notably, RB annihilates into the most stable B by a low barrier outer shift (dashed black line), while the R‐1 to B transition requires an inner interface shift with a higher energy barrier (see dashed red line). Instead, R‐1 can annihilate into R or S by an outer interface shift, which explains the tendency to observe these three phases together (see Figure S1b, Supporting Information). The larger abundance of the S phase compared to R may suggest its better relative stability and lower ground state energy. Similarly, the rare appearance of V could point to a less stable stacking energy, which adds up to a low barrier path to RB and then B phase. The latter explains the annihilation dynamics shown in Figure S1 (Supporting Information), where the V domains embedded in RB domains eventually annihilate to the B polytype. Note that the observed trends in the relative abundance of different polytypes may also be influenced by kinetic factors during exfoliation.

## 6 Deterministic Raman Identification

**Figure 4:**
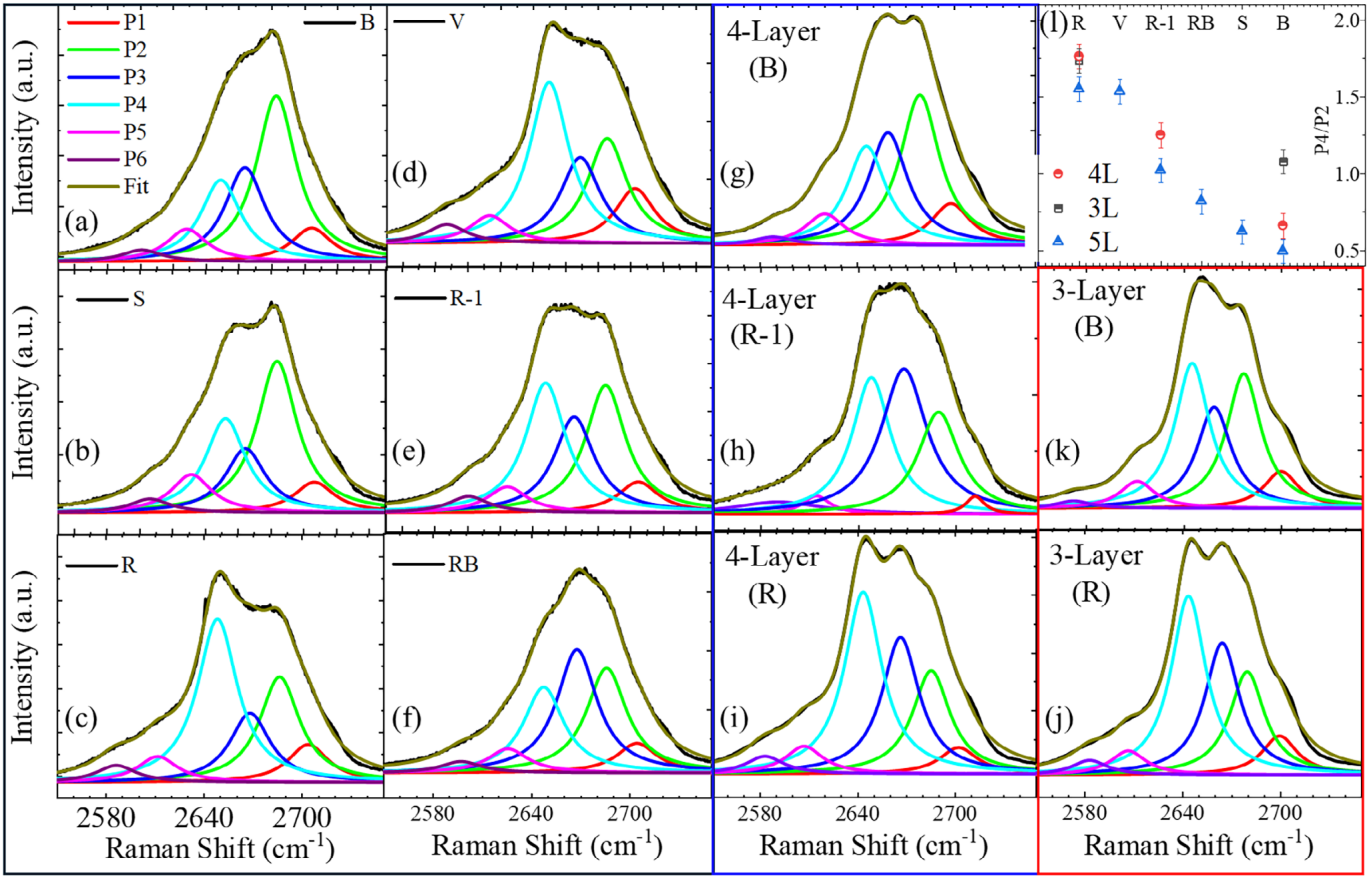
Analysis of 2D Raman peaks under red laser illumination. a–f) six penta layers polytypes, g–i) tetra‐layer three polytypes, and j,k) tri‐layer polytypes, respectively. All spectra are fitted with six Lorentzian functions within the 2500–2800 cm^−1^ range. l) The ratio of the integrated intensity of peak 4 (cyan) to peak 2 (green).

After identifying the Raman spectrum of each polytype, we use the intensity variations in the 2D peak under red laser excitation as a useful tool to distinguish the polytypes. In general, the 2D peak involves multiple K to K’ double resonance scattering processes, resulting in a large number of overlapping Lorentzians. In bilayers, for example, the four Lorentzians observed were attributed to three dominant resonance processes,^[^ ^29^ ^]^ although four independent scattering processes were previously suggested.^[^ ^30^, ^31^ ^]^ For trilayers the number of dominant Lorentzians grows to six out of 15 allowed.^[^ ^13^ ^]^ Similarly, we find that the penta layers 2D peaks can also be fitted quite accurately using at least six Lorentzians (see **Figure** **4**a–f). Here, each spectrum is fitted freely within the 2500–2800 cm^−1^ range, and the intensity of the P2 and P4 that accumulate most of the scattered photons is compared. We find the intensity of the high‐energy resonance (green P2 curve) to scale with the number of monomers (2 for R and 5 for B), while the low‐energy P4 resonance intensity (cyan curve) scales with the number of dimers (4 and 0, respectively). While adding more components (seven or eight Lorentzians) to the fitting slightly alters the individual peak intensities, it does not significantly improve the overall fit quality (Section S3, Supporting Information). Importantly, the integrated intensity ratios of the most prominent peaks remain consistent across different samples for each fitting approach, as shown in Section S4 (Supporting Information).

While an accurate calculation of the full Raman spectra is challenging and goes beyond the scope of this work, we report a complete data set of the trilayer and tetralayer polytypes' spectra. The ratio P4/P2 of all the possible polytypes is shown in Figure 4l.

## 7 Surface Potential Variations

**Figure 5:**
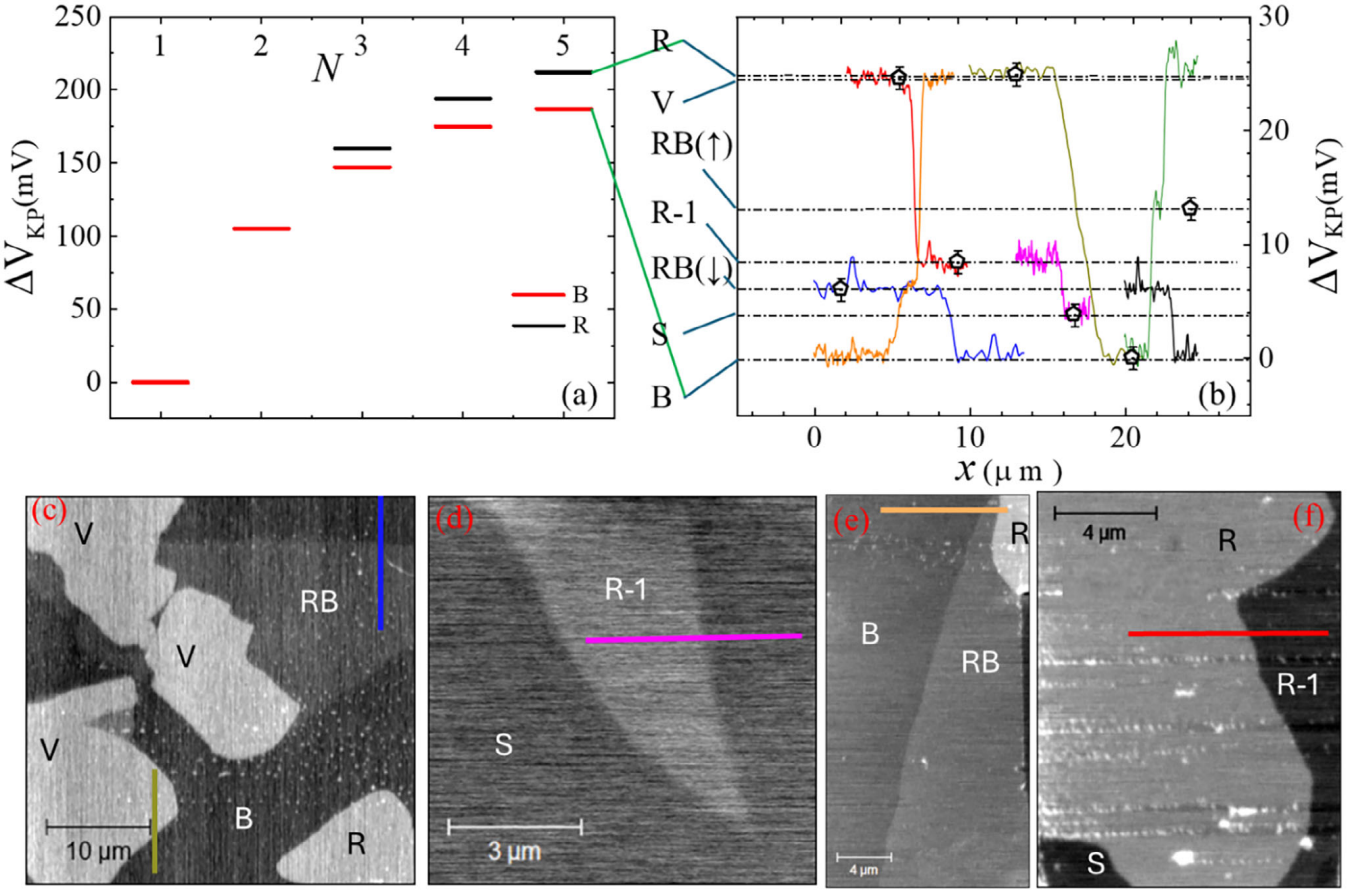
Polytypes surface potential. a) Surface potential difference ΔV_KP_ versus the number of layers for the B and R polytypes. b) Line cuts of the potential across peta‐layer polytype boundaries, including the up and down polar configuration of the RB phase. Open pentagons mark the average value. c–f) Maps of four different samples that were used to extract the line cuts.

An alternative, more localized approach to distinguishing polytypes is to measure variations in their work function. To achieve this, we map the surface potential of different polytypes using Kelvin probe atomic force microscopy (KPFM). **Figure** **5**a presents the evolution of surface potential as a function of layer number relative to the monolayer potential, defined as ΔV_KP_ = V_KP_ (N‐layers) − V_KP_ (1‐layer). The potential values for B and R stacking are shown in red and black, respectively (see detailed topography and potential maps in Figure S8, Supporting Information). The observed increasing trend of ΔV_KP_ with layer number and its asymptotic saturation to the bulk value aligns with previous reports on the work function evolution.^[^ ^32^, ^33^ ^]^

Notably, surface potential values in monolayer and bilayer graphene are strongly influenced by doping due to the steep band dispersion, which shifts the Fermi level away from the Dirac point.^[^ ^32^, ^33^, ^34^ ^]^ Our gate‐dependent surface potential measurements, conducted after AFM cleaning and under inert conditions, indicate a doping level of 5 × 10^12^ holes cm−^2^ (see Figure S8 and Section S6, Supporting Information). However, in polytypes thicker than three layers, the surface potential differences become largely independent of doping, consistent with previous reports.^[^ ^34^ ^]^

Figure 5b illustrates the surface potential differences between all penta‐layer polytypes, measured relative to the B phase. These work function variations provide an additional means of distinguishing polytypes, as well as identifying the out‐of‐plane orientation of the polar configurations. Notably, we find an intrinsic RB polytype polarization of (V_KP_(P^RB^↑)–V_KP_(P^RB^↓))/2 = 3±1 mV (Section S5, Supporting Information), about half of the intrinsic polarization of the polar polytype in four layers.^[^ ^1^, ^35^, ^36^, ^37^ ^]^ Due to their very low abundance, we could not reveal the R‐1 polarization yet.

While the absolute surface potential values can vary by up to 200 mV between experiments, the relative potential steps between polytypes remain consistent. Moreover, these measurements are independent of polytype dimensions, the AFM tool used, or the tip type.

## 8 Conclusion

This study reports the deterministic identification of all six polytypes in penta‐layer graphene obtained from naturally exfoliated graphene on 90 nm Si/SiO_2_ substrate using Raman spectroscopy under red light illumination. Polytypes that break [I] symmetry can be further distinguished through their SHG response, though caution is required when analyzing the symmetric R phase. Additionally, a more localized method for polytype distinction is provided by surface potential measurements, which also reveal intrinsic polarization orientations.

The observed polytype distribution suggests greater stability for the B, R, and S polytypes compared to the V, RB, and R‐1 phases. Furthermore, the overall polytype abundances and their annihilation dynamics are influenced by the mobility of boundary strips. Notably, boundary strip motion in surface layers appears to be more facile than shifts occurring within internal interfaces. While the present experiments cannot probe the polytypes’ transition dynamics in real‐time, they demonstrate the low barriers involved and the need to prevent spontaneous annihilation for a more detailed exploration of the unique properties of polytypes such as V, RB, and R‐1. Further research into these delicate structural transitions will support the development of multiferroic “slidetronic” devices, which leverage interlayer van der Waals sliding for functional applications.^[^ ^24^, ^25^ ^]^

## 9 Experimental Section

The samples were prepared by exfoliating natural graphite (NGS) onto 90 nm‐thick Si/SiO2 wafers using the standard scotch tape technique. The Si/SiO_2_ wafer was first cleaned in an ultrasonic bath using acetone, followed by isopropyl alcohol (IPA). After drying with nitrogen gas, the wafer was baked on a hot plate at 180 °C for 5 mins toremove any residual solvents or moisture. Oxygen plasma treatment was then performed to further clean the surface and improve adhesion. Immediately after plasma cleaning, freshly cleaved graphite flakes on Scotch tape were pressed onto the wafer surface. The tape was pressed firmly using a fingertip to promote contact, and then carefully removed. Thin graphene flakes were subsequently identified using optical microscopy. Optical contrast and AFM were utilized to determine the number of layers. Raman spectroscopy measurements were conducted using a WITEC alpha300 Apyron confocal microscope equipped with a UHTS 300 mm focal length spectrometer. A 532 nm laser with a 300 lines mm^−1^ grating was employed for large area scans, while a 633 nm laser with a 1200 lines mm^−1^ grating was used for point spectra. For large area scans, a step size of 200 nm was used and an average laser power of ≈5 mW was maintained. Integration times shorter than 1 s were used to prevent laser‐induced heating. Point spectra acquired with the 633 nm laser utilized a 120 s exposure time and four accumulations to enhance the signal‐to‐noise ratio.

For the statistical data in Figure 3a, the first Raman scan recorded immediately after exfoliation was taken as the baseline for each flake. This ensured that all initially observed states, including metastable configurations, were accounted for. Subsequent scans were used only to track transitions or relaxation and were not included in the statistics. Hence, the relaxation process does not influence the statistical distribution shown in Figure 3a.

SHG measurements were performed using a linearly polarized 1064 nm ultrafast fiber laser (Rainbow 1064, 15 ns pulse width, 10 MHz repetition rate). The laser beam was focused onto the sample at normal incidence using a 50 × objective lens with a numerical aperture of 0.55. The emitted signal was collected through the same lens in reflection geometry and detected by the UHTS 300 spectrometers.

AFM topography and KPFM measurements were carried out using a PARK NX‐HIVAC system in a nitrogen gas environment at 100 mbar pressure. A PPP‐EFM metal‐coated tip with a mechanical resonance frequency of ≈75 kHz and a spring constant of 3 N m^−1^ was used. During KPFM measurements, the tip operated in non‐contact modes with a cantilever amplitude ranging between 10 and 30 nm with a scan rate of 0.25 Hz. The AFM topography and KPFM signals were obtained separately in a two‐pass measurement approach. In the first pass, topography was recorded. The second pass involved lifting the tip an additional 7 nm to record KPFM DC potential under a bias voltage of 1.5 V AC at a frequency of 1.5–3 kHz. The sideband KPFM mode was utilized to achieve a more precise characterization of local potential variations

## Conflict of Interest

The authors declare no conflict of interest.

## Supporting information



Supporting Information

## Data Availability

The data that support the findings of this study are available from the corresponding author upon reasonable request.
