# Supplementary material for: Detecting the Six Polytypes of Five‐Layer Graphite
Source: Adv Mater. 2025 Sep 2;37(47):e09947. doi: 10.1002/adma.202509947 (PMC12651134; doi:10.1002/adma.202509947)
Supplement: Supplementary file 1 — Supporting Information [file ADMA-37-e09947-s001.docx]

Supplementary Information

**Detecting the Six Polytypes in Five-Layer Graphene**

Nirmal Roy, Shaked Amitay, Simon Salleh Atri, Harel Kabla, Oren Ben Moshe, Moshe Ben Shalom

School of Physics and Astronomy, Tel Aviv University, Tel Aviv, Israel

Contents

[**SI.1 Stability of polytypes.** 2](#_Toc204358115)

[**SI.2 Additional samples** 3](#_Toc204358116)

[**SI.3 Justification for Using Six Lorentzian Peaks in Raman Fitting:** 5](#_Toc204358117)

[**SI.4 Additional fititing approach of 2D Raman peaks:** 5](#_Toc204358118)

[**SI.5 Electric polarization in RB phase** 8](#_Toc204358119)

[**SI.6 Surface potential distribution of multilayer sample** 9](#_Toc204358120)

[**SI.7 Tight binding calculations.** 10](#_Toc204358121)

[References 12](#_Toc204358122)

**List of Figures:**

[Figure S.1 Shrinking of metastable polytypes 4](#_Toc204879330)

[Figure S.2 Characterization of polytype configuration 5](#_Toc204879331)

[Figure S.3: (a–e) 2D Raman spectra of the V polytype 6](#_Toc204879332)

[Figure S.4: Analysis of 2D Raman peaks with six Lorentzian functions. 7](#_Toc204879333)

[Figure S.5 Analysis of 2D Raman peaks with seven Lorentzian functions 8](#_Toc204879334)

[Figure S.6 Analysis of 2D Raman peaks with eight Lorentzian functions 9](#_Toc204879335)

[Figure S.7 B, R, and RB polytypes. 10](#_Toc204879336)

[Figure S.8 (a) Lines cuts of the surface potential 11](#_Toc204879337)

[Figure S.9 Schematic representation 12](#_Toc204879338)

**List of Tables:**

Table S1: Polytype Identification Process………………………………………………………………….2

Table S1: Polytype Identification Process


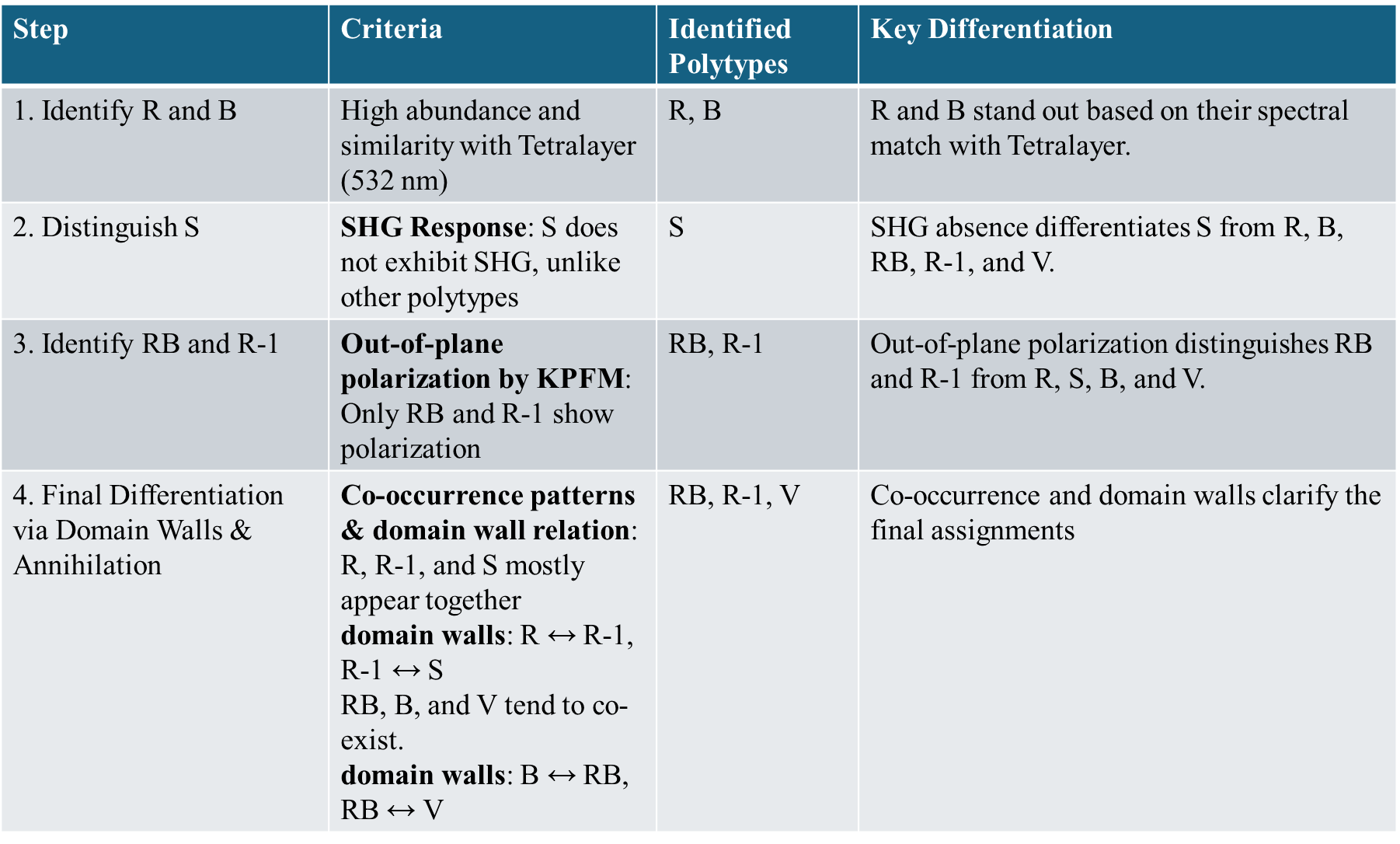


## **SI.1 Stability of polytypes.**

To examine the stability among the metastable phases of Penta layer polytypes, we performed Raman mapping of domains under green laser excitation over time on several samples. We find that R, RB, V, R-1 polytypes tends to shrink in time into the most stable B polytype, while the R-1 phase shrink into S or R phases. Figure SI.1 presents three distinct samples and their corresponding 2D Raman maps. Each map in the panels (from left to right) was acquired at one-month intervals, highlighting the evolution of domains: (a) B, RB, V, R; (b) R-1, R, S; and (c) B, RB, R. For instance, in panel (a), the polytypes V, RB, and R, which share domain walls with B, gradually shrink or disappear, allowing the B configuration to expand. Similarly, in panel (b), the domain wall between R and R-1 evolves and reorients, resulting in the expansion of the R domain relative to R-1. In panel (c), the RB polytype vanishes entirely, while significantly expanding the B region. These observations may suggest that B, R, and S polytypes are relatively more stable or kinetically favored in pentalayer graphene, while the other polytypes appear less stable or metastable and tend to evolve toward these configurations over time. We note that between the consecutive Raman scans shown in Fig.SI.1, the samples were also subjected to AFM cleaning, KPFM, and SHG measurements, which may have introduced perturbations that help depin domain walls and promote relaxation toward more stable states. However, we also observed spontaneous changes even without such treatments, suggesting that local strain and pinning conditions, as well as external stimuli, may play an equally important role.


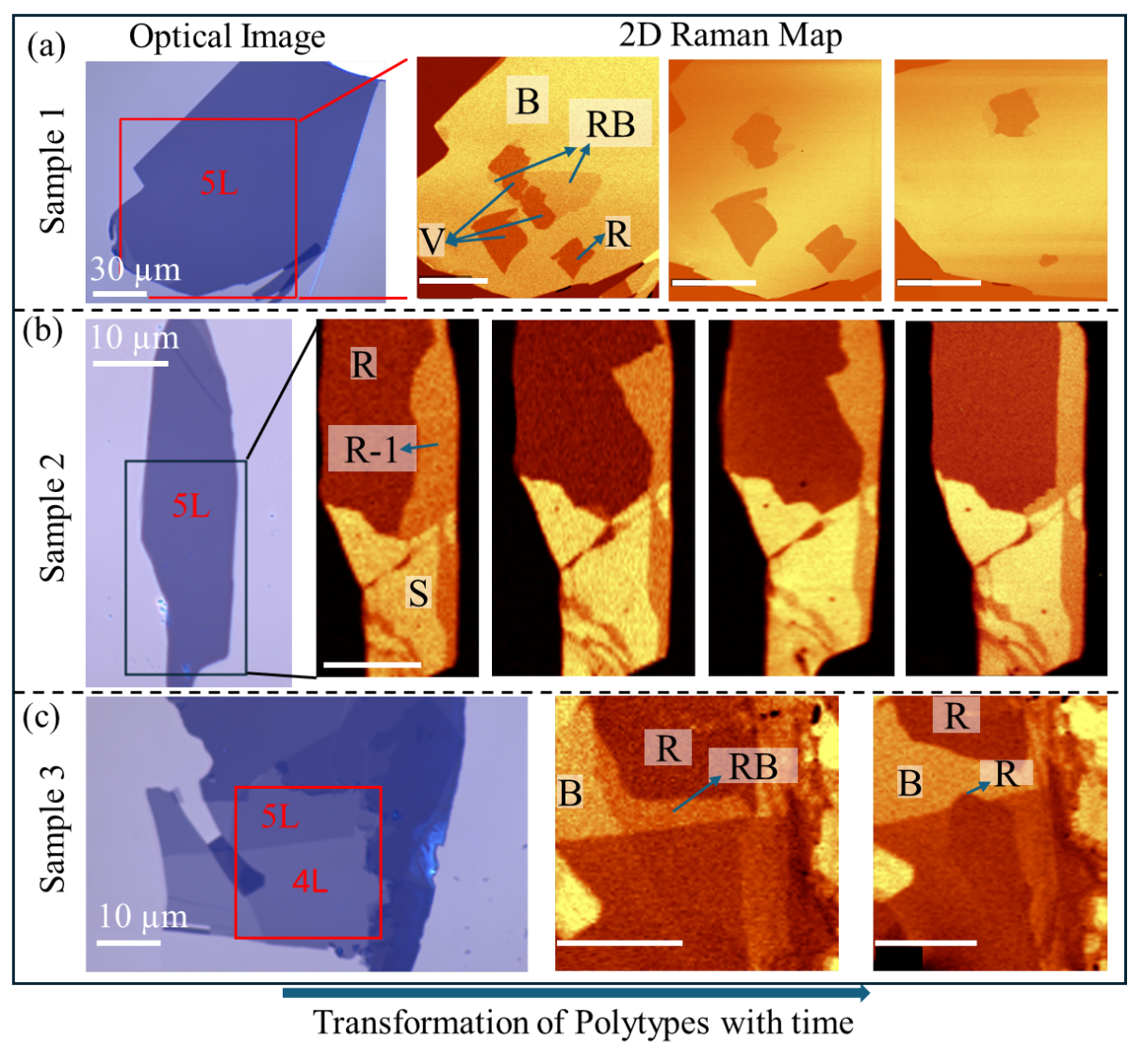


Figure S.1 Shrinking of metastable polytypes in time (a) R, V and RB to B (b) R-1 to R (c) RB to B

## **SI.2 Additional samples**

Figure S.2 shows an example of Penta layer graphene (Sample 4) with R-1 and S polytypes, and Sample 5 with R, R-1 and B polytypes. The optical image and Raman maps of the marked red rectangle area are shown in Fig. S.2a and c. The surface potential map in Fig. S.2b and single-point Raman spectra using red laser excitation in Fig. S.2d, taken inside these two domains, confirm the distinct signatures of the R-1 and S polytypes. Similar characterization is presented for sample 5 in Fig. S.2 (e-h)


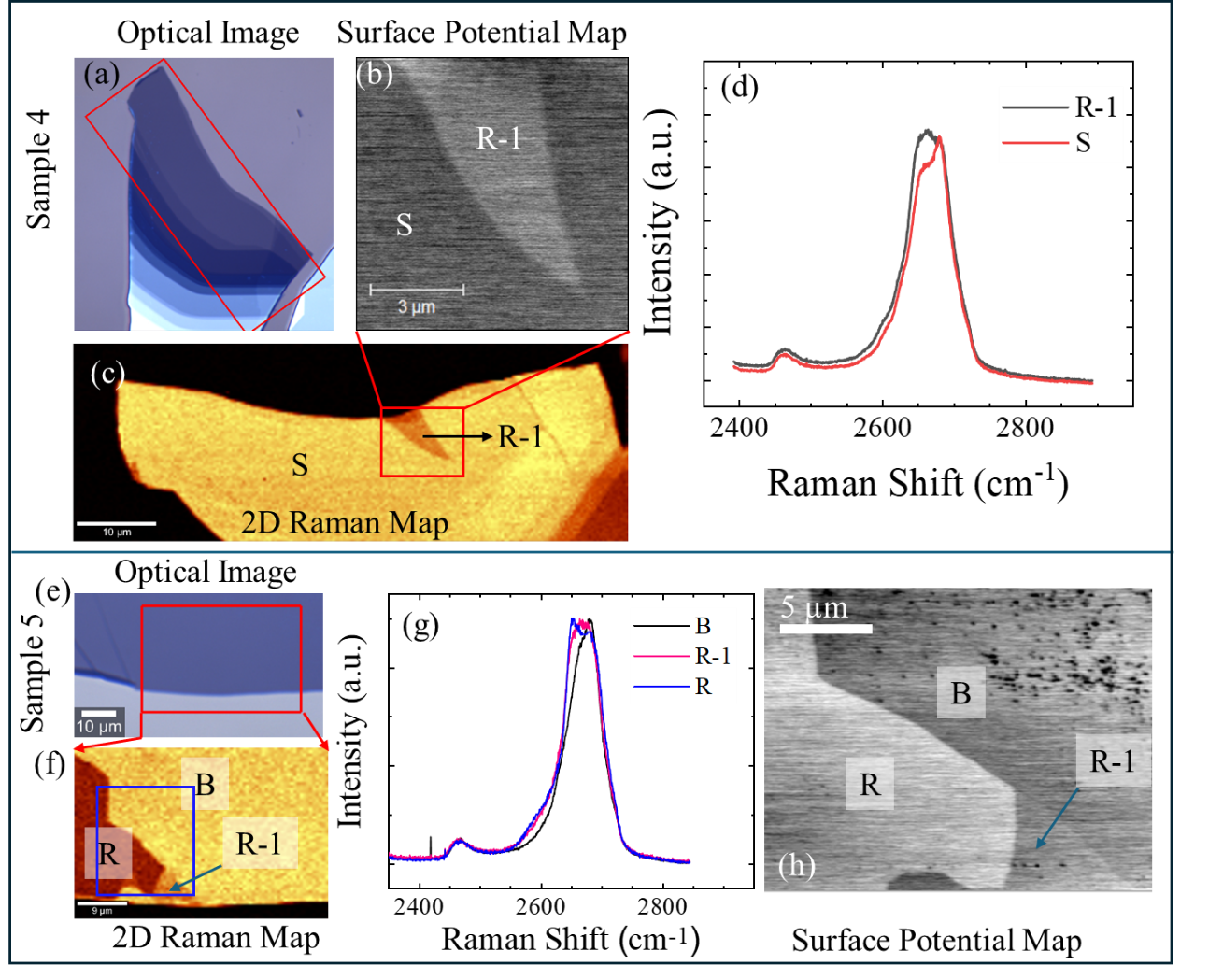


Figure S.2 Characterization of polytype configurations in Samples 4 and 5. Sample 4: R-1 and S polytypes. (a) Optical image; (b) Surface potential map; (c) Raman intensity map; (d) Raman spectra acquired using red laser excitation. The surface potential profile shown in Figure 4b of the main text was extracted from this sample. Sample 5: R, R-1, and B polytypes. (e) Optical image; (f) Raman intensity map; (g) Raman spectra using red laser excitation; (h) Surface potential map of marked blue rectangular region in (f).

## **SI.3 Justification for Using Six Lorentzian Peaks in Raman Fitting:**

Our choice of fitting six Lorentzian (although there are clearly many more resonances involved) is attempting to capture the data with minimal complexity. Fig. S.3f shows the misfit χ^2^ values as a function of the number of fitted Lorentzian (panel a-e). We find a substantially better fit for six versus five, but not much improvement beyond that – see panel f in Fig. 3.S.


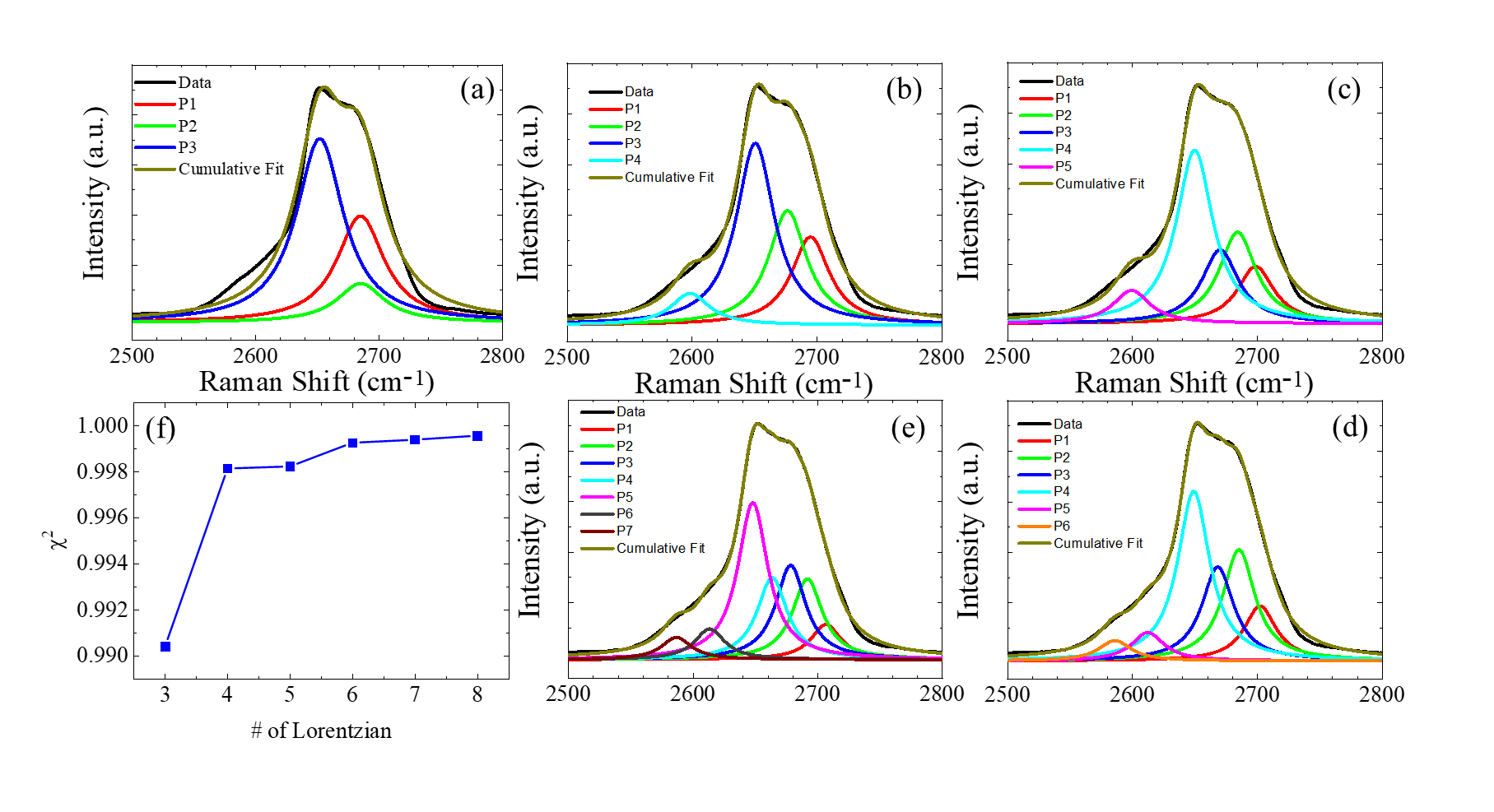


Figure S.3: (a–e) 2D Raman spectra of the V polytype under red laser illumination, fitted with three, four, five, six, and seven Lorentzian components, respectively. (f) Variation of the goodness-of-fit (χ^2^) as a function of the number of Lorentzian components.

## **SI.4 Additional fittings approach of 2D Raman peaks:**

To further demonstrate the reproducibility and robustness of the Raman analysis presented in the main text, Figure (S.4-S.6) shows the additional data sets of 2D Raman spectra of penta-layer graphene polytypes, measured under red laser excitation. All spectra were systematically fitted with six (Fig.S4), seven (Fig.S5) and eight (Fig.S6) Lorentzian components within the 2500–2800 cm⁻¹ range. Adding more Lorentzian to the fitting procedure does not improve the fitting much as mentioned in the previous section. The integrated intensity ratios of the dominant peaks are shown in Fig. S4g (P4/P2), Fig. S5g (P5/P2), and Fig. S6g (P5/P2), corresponding to six-, seven-, and eight-component Lorentzian fittings, respectively. While the values of integrated intensity ratios may vary with the number of Lorentzian components used; these ratios remain highly consistent across different samples when the same fitting method is applied. This internal consistency within each fitting scheme highlights the robustness and reproducibility of the spectral fingerprints, can be used for distinguishing different stacking polytypes.


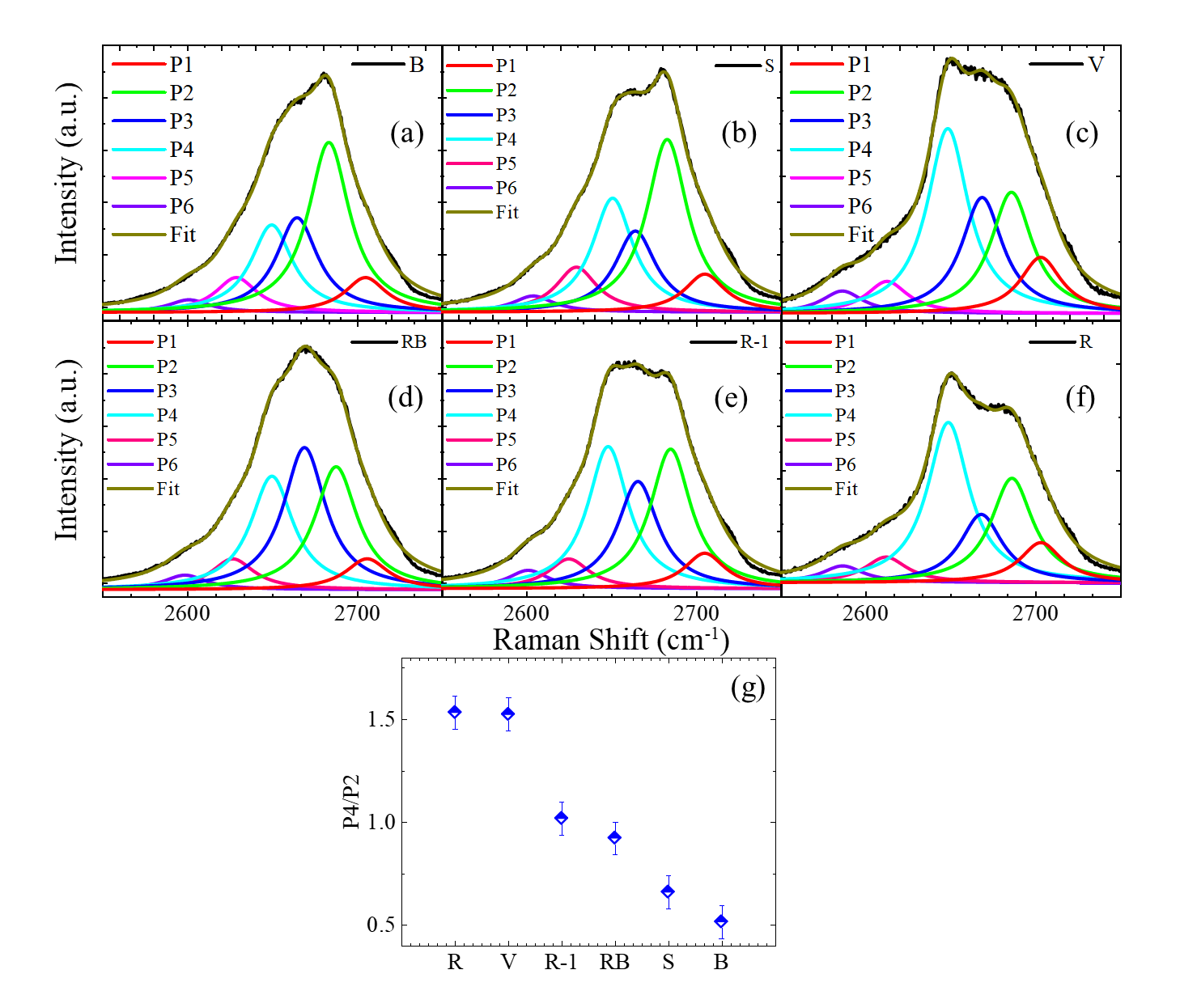


Figure S.4: Analysis of 2D Raman peaks with six Lorentzian functions: 2D Raman peaks under red laser illumination (a)-(f) six penta-layers polytypes. All spectra are fitted with six Lorentzian functions within the 2500-2800 cm⁻¹ range. (g) The ratio of the integrated intensity of peaks 4 (cyan) to peak 2 (green).


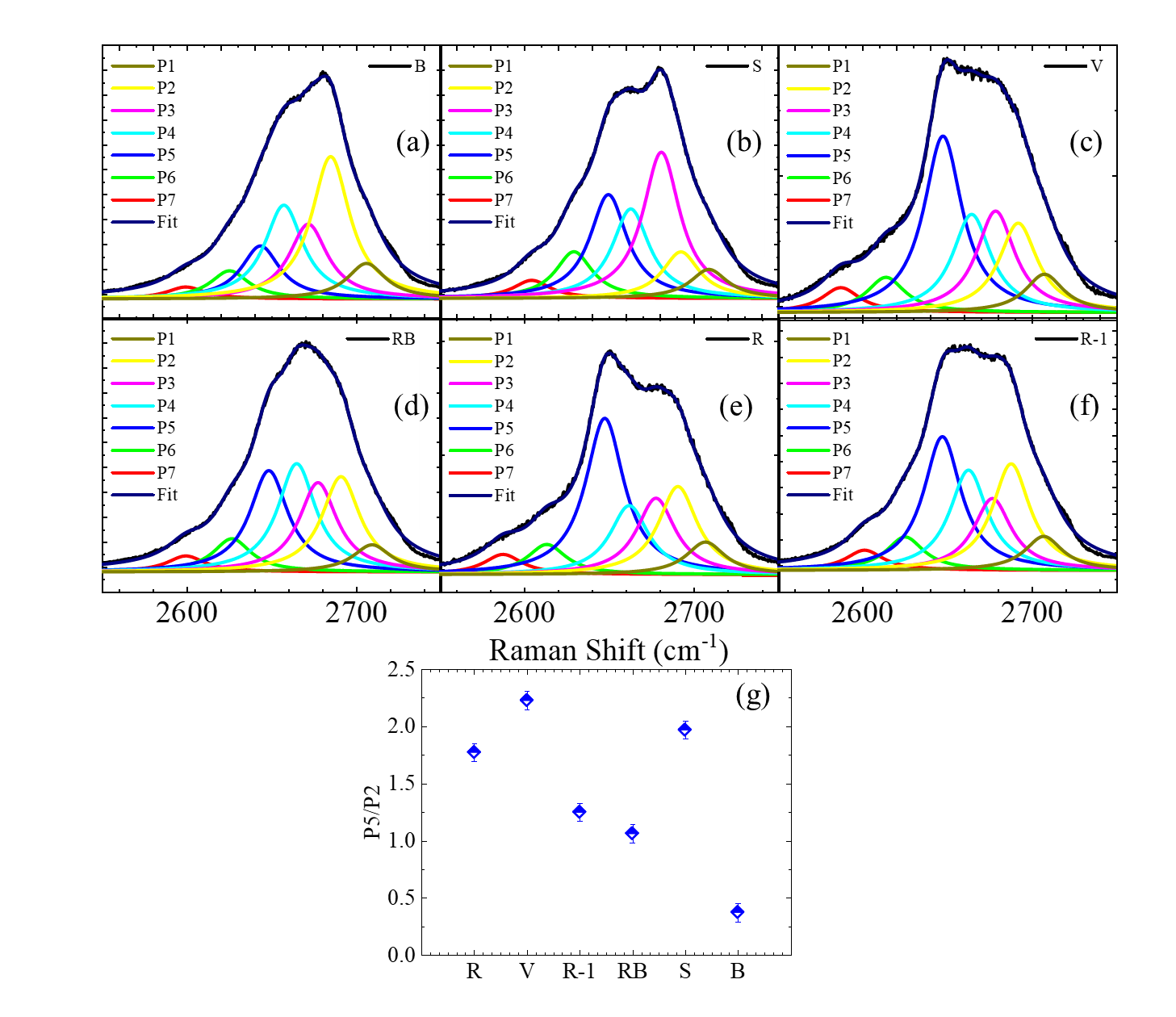


Figure S.5 Analysis of 2D Raman peaks with seven Lorentzian functions: 2D Raman peaks under red laser illumination (a)-(f) six penta-layers polytypes. All spectra are fitted with seven Lorentzian functions within the 2500-2800 cm⁻¹ range. (g) The ratio of the integrated intensity of peaks 5 (blue) to peak 2 (yellow).


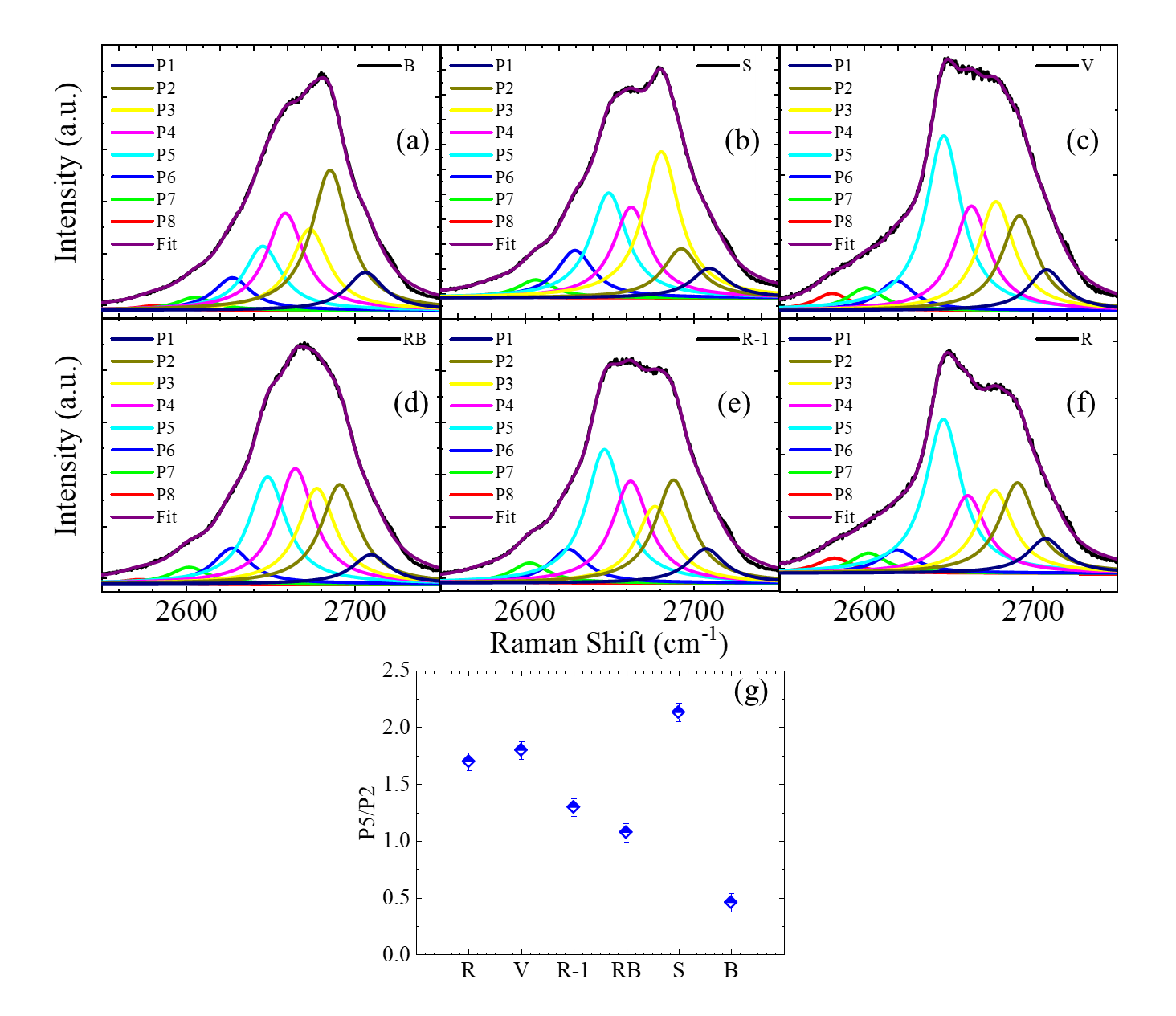


Figure S.6 Analysis of 2D Raman peaks with eight Lorentzian functions: 2D Raman peaks under red laser illumination (a)-(f) six penta-layers polytypes. All spectra are fitted with eight Lorentzian functions within the 2500-2800 cm⁻¹ range. (g) The ratio of the integrated intensity of peaks 5 (cyan) to peak 2 (dark green).

## **SI.5 Electric polarization in RB phase**

Two additional samples (6 and 7) with R, RB, and B polytypes are presented in Fig. S.7. The optical images, 2D Raman maps using green laser, and surface potential maps of the selected regions are shown in Figs. S.7(a-c) and S.7(d-f), respectively. The 2D Raman spectra of the RB polytypes (recorded using red laser excitation), shown in Fig. S.7(g), exhibit identical line shape across both samples. However, in the surface potential maps, the line cut along the dashed lines in Figs. S.7(c) and S.7(f) through the B, RB, and R regions reveals a 7 mV difference between the RB domains of the two samples, confirming the internal electrical polarization of the RB polytypes as (V_KP_(P^RB^↑) – V_KP_(P^RB^↓))/2 = 3±1 mV.


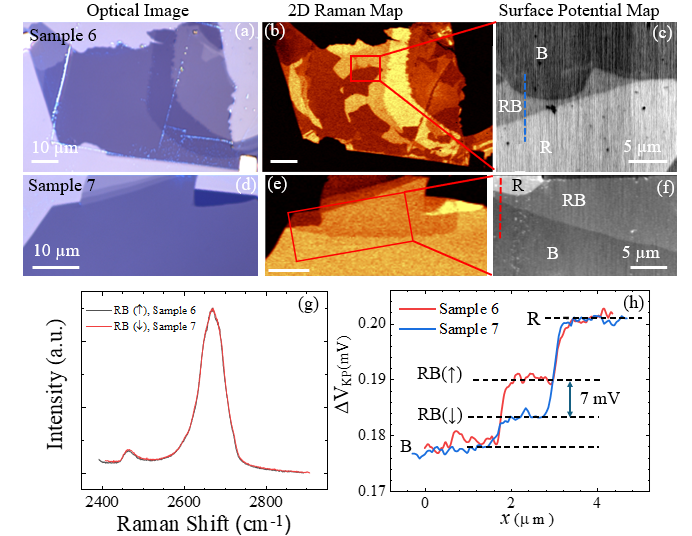


Figure S.7 B, R, and RB polytypes. (a–c) Sample 6: (a) Optical image, (b) Raman map, and (c) SP map of the region outlined in red. (d–f) Sample 7: (d) Optical image, (e) Raman map, and (f) SP map of the region outlined in red. (g) Identical Raman 2D spectra from the RB regions of samples 6 and 7. (h) SP line profiles along the blue dashed line in (c) and the red dashed line in (f), showing a potential difference of 7 mV between the RB regions of the two samples.

## **SI.6 Surface potential distribution of multilayer sample**

To compare the variation in work function with the number of graphene layers, we measured the surface potential distribution across a multilayer graphene sample with monolayer to penta layer graphene flakes, as shown in Fig. S.8. Figures S.8b and S.8d present the measured surface potential maps correspond to the marked regions in Fig. S.8c. The absolute values of surface potential along line cuts across different layers (Figs. S.8b and S.8b) are displayed in Fig. S.8a. Additionally, line cuts from different samples are included to capture the potential steps of the B and R polytypes up to Penta layer graphene.

To assess the doping level following surface cleaning, we measured the doping-dependent surface potential distribution across various layers. Figure S.8e illustrates the average surface potential variation up to penta layer graphene as a function of the bottom Si gate voltage (±8.5 V). The substantial variation in surface potential observed in layers up to Tri-layer graphene is attributed to intrinsic doping. However, for five layers, the surface potential remains largely independent of doping.

Figure S.8f shows the difference in surface potential between monolayer and bilayer graphene within a bottom gate voltage range of ±8.5 V. The slope of this curve is compared with theoretical density functional theory (DFT) calculations^1^ in Fig. S.8g, and the best-matching slope provides an estimate of the doping level of 5×10^12^ holes/cm^2^ in our sample. In general, an intrinsic doping concentration of approximately ~ 4×10^12^ holes/cm^2^ is observed in exfoliated graphene on a Si/SiO_2_ substrate^1–3^.


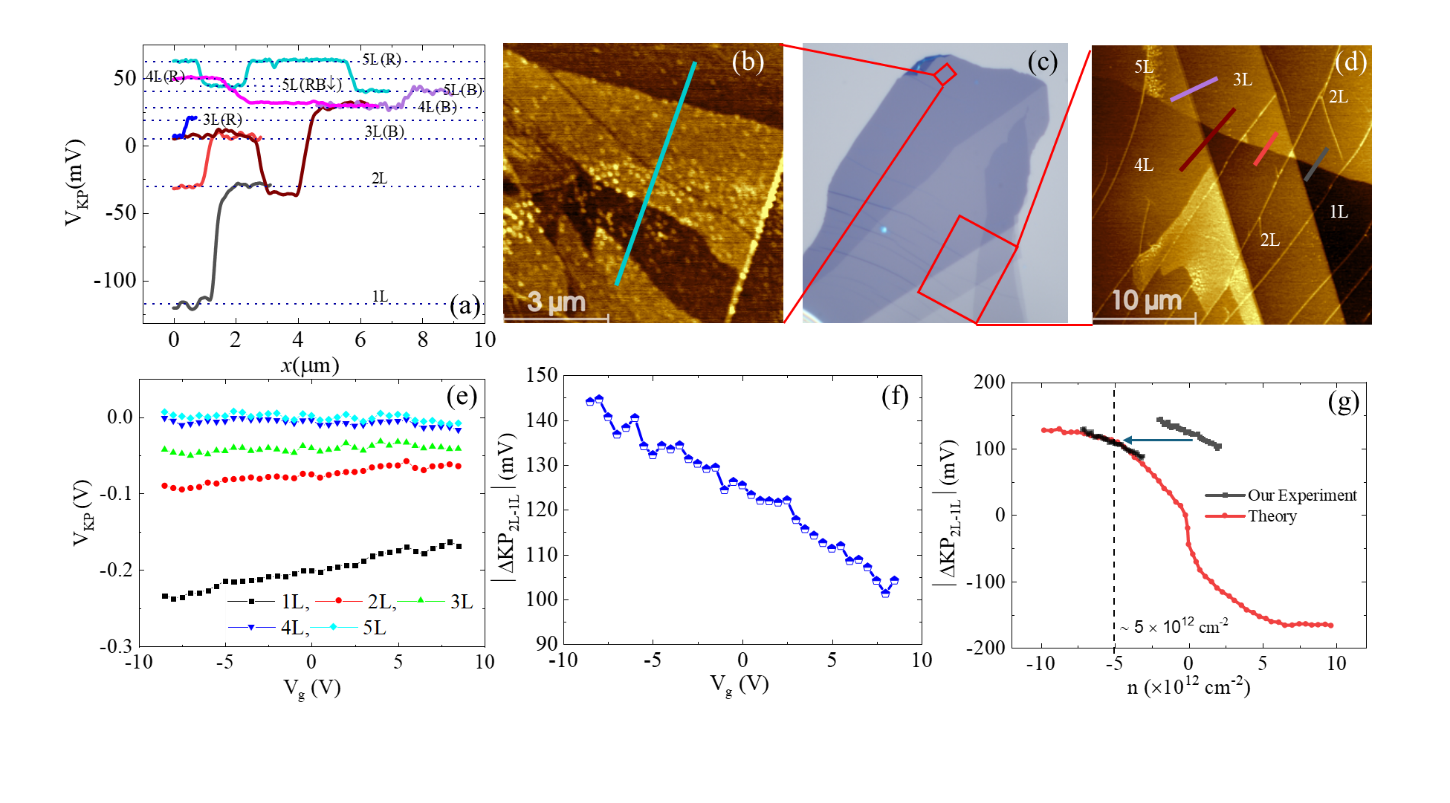


Figure S.8 (a) Lines cuts of the surface potential maps shown in (b, d). (c) optical microscope image. (e) Surface potential variations as a function of the applied gate voltage (f). The potential difference between a bilayer and a monolayer graphene. (g) Comparison of the curve in (f) with the theoretical response expected^1^. The fitted slope indicates a doping level of 5×10^12^ holes/cm^2^.

## **SI.7 Tight binding calculations.**

Our single-particle band structure tight binding calculations^4^ use the Slonczewski-Weiss-McClure (SWM) hopping parameters represented in Figure S.9 with their respective values^5^.We follow the convention of using an opposite sign for γ_4_ in its corresponding matrix elements ^6^.


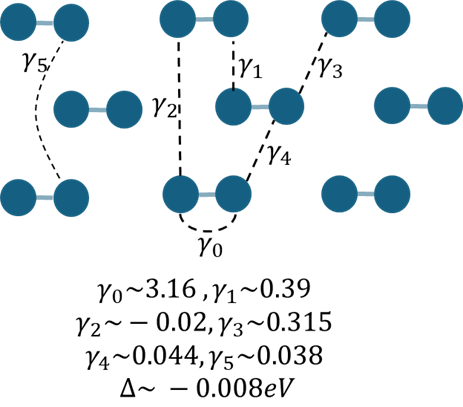


Figure S.9 Schematic representation of the SWM hopping parameters of Graphite, with their corresponding values.

# References

1. Ziegler, D. *et al.* Variations in the work function of doped single- and few-layer graphene assessed by Kelvin probe force microscopy and density functional theory. *Phys Rev B Condens Matter Mater Phys* **83**, (2011).

2. Yu, Y. J. *et al.* Tuning the graphene work function by electric field effect. *Nano Lett* **9**, 3430–3434 (2009).

3. Ji, E. *et al.* Substrate effect on doping and degradation of graphene. *Carbon N Y* **184**, 651–658 (2021).

4. McCann, E. & Koshino, M. The electronic properties of bilayer graphene. *Reports on Progress in Physics* **76**, 056503 (2013).

5. Dresselhaus, M. S. & Dresselhaus, G. Intercalation compounds of graphite. *Adv Phys* **30**, 139–326 (1981).

6. Partoens, B. & Peeters, F. M. From graphene to graphite: Electronic structure around the K point. *Phys Rev B Condens Matter Mater Phys* **74**, (2006).
